# Examining Responsiveness to an Incentive-Based Mobile Health App: Longitudinal Observational Study

**DOI:** 10.2196/16797

**Published:** 2020-08-10

**Authors:** Jacob Brower, Monica C LaBarge, Lauren White, Marc S Mitchell

**Affiliations:** 1 Smith School of Business Queen's University Kingston, ON Canada; 2 Carrot Insights Inc Toronto, ON Canada; 3 School of Kinesiology Western University London, ON Canada

**Keywords:** mHealth, behavioral economics, public health, incentives, mobile apps, mobile phone

## Abstract

**Background:**

The Carrot Rewards app was developed as part of a public-private partnership to reward Canadians with loyalty points for downloading the app, referring friends, completing educational health quizzes, and health-related behaviors with long-term objectives of increasing health knowledge and encouraging healthy behaviors. During the first 3 months after program rollout in British Columbia, a number of program design elements were adjusted, creating observed differences between groups of users with respect to the potential impact of program features on user engagement levels.

**Objective:**

This study examines the impact of reducing reward size over time and explored the influence of other program features such as quiz timing, health intervention content, and type of reward program on user engagement with a mobile health (mHealth) app.

**Methods:**

Participants in this longitudinal, nonexperimental observational study included British Columbia citizens who downloaded the app between March and July 2016. A regression methodology was used to examine the impact of changes to several program design features on quiz offer acceptance and engagement with this mHealth app.

**Results:**

Our results, based on the longitudinal app use of 54,917 users (mean age 35, SD 13.2 years; 65.03% [35,647/54,917] female), indicated that the key drivers of the likelihood of continued user engagement, in order of greatest to least impact, were (1) type of rewards earned by users (eg, movies [+355%; *P*<.001], air travel [+210%; *P*<.001], and grocery [+140%; *P*<.001] relative to gas), (2) time delay between early offers (−64%; *P*<.001), (3) the content of the health intervention (eg, healthy eating [−10%; *P*<.001] vs exercise [+20%, *P*<.001] relative to health risk assessments), and (4) changes in the number of points offered. Our results demonstrate that reducing the number of points associated with a particular quiz by 10% only led to a 1% decrease in the likelihood of offer response (*P*<.001) and that each of the other design features had larger impacts on participant retention than did changes in the number of points.

**Conclusions:**

The results of this study demonstrate that this program, built around the principles of behavioral economics in the form of the ongoing awarding of a small number of reward points instantly following the completion of health interventions, was able to drive significantly higher engagement levels than those demonstrated in previous literature exploring the intersection of mHealth apps and financial incentives. Previous studies have demonstrated the presence of incentive matters to user engagement; however, our results indicate that the number of points offered for these reward point–based health interventions is less important than other program design features such as the type of reward points being offered, the timing of intervention and reward offers, and the content of the health interventions in driving continued engagement by users.

## Introduction

If modifiable chronic disease risk factors (eg, physical inactivity, unhealthy eating) were eliminated, 80% of both ischemic heart disease and type 2 diabetes and 40% of cancers could be prevented [1]; consequently, modest population-level improvements can make a big difference. For instance, a 1% reduction in the proportion of Canadians accumulating less than 5000 daily steps would yield Can $2.1 billion (US $1.615 billion) per year in health care system savings [2]. Such health behaviors, though, are notoriously difficult to stimulate and sustain, with persistent global overweight and obesity rates providing cases in point [1]. The World Health Organization [3] and others [4] suggest that broader socioecological solutions with interventions delivered at multiple levels (eg, individual, community, societal) are needed to address this issue. At the individual level, smartphones have revolutionized health promotion [5]. Their pervasiveness (eg, 1 billion global smartphone subscriptions expected by 2022) [6] and rapidly evolving functionalities (eg, built-in accelerometers, geolocating capabilities, machine learning techniques) have made it easier to deliver more timely and personalized health interventions on a mass scale.

### Mobile Health Apps

The smartphone-based mobile health (mHealth) app market has grown steadily in recent years. In 2017, for example, there were 325,000 mHealth apps available on all major app stores, up by 32% from the previous year [7]. The number of mHealth app downloads also increased by 16% from 2016 to 2017 (3.2 to 3.7 billion) [7]. Although both supply (apps published in stores) and demand (app downloads) is growing, low engagement (with engagement measured as repeated usage, consistent with behavioral science approaches [8,9]) has resulted in small effect sizes and presented hurdles for financial sustainability continues to be a challenge for the industry [10-13]. For instance, 90% of all mHealth apps are uninstalled within 30 days, and 83% of mHealth app companies have fewer than 10,000 monthly active users, a standard industry engagement metric [14]. Systematic reviews of controlled studies on this topic suggest that tailoring content to individual characteristics, regularly updating apps, and incorporating a range of behavior change techniques, for example, may boost engagement [10-13]. To date, however, evaluations of only a few mHealth apps out of the thousands in the app stores have been published in peer-reviewed scientific journals [12]. To better elucidate the conditions under which mHealth app interventions are likely to succeed in real-world settings, more applied research is needed [5]. Traditional randomized controlled trials can be difficult to conduct in a fast-paced digital health environment, but mHealth has benefited from innovative approaches which attempt to determine causal mechanisms for outcomes of intervention effectiveness through methods such as microrandomized trials, factorial designs, and quasiexperimental designs such as pre-post, inverse roll-out, and interrupted time series [15]. Given the widespread proliferation of mHealth apps, evaluation methods that examine what does or does not work in the field, even when not employing methodologies that may generate interpretations of causality, may still contribute to our understanding of the contextual (eg, population characteristics) and program (eg, intervention design) factors that impact engagement, which ultimately influence the effectiveness both in terms of the financial cost of interventions as well as measurable impacts on health. This study, therefore, responds to a call in the literature to create a more comprehensive understanding of contextual and program factors that may impact engagement.

### Study Context

The Carrot Rewards app (Carrot), created by a private company with support from the Public Health Agency of Canada [16], presents a unique research opportunity to explore the effects of some of these factors. Carrot was a Canadian app (ie, 1 million downloads, 500,000 monthly active users) grounded in behavioral economics, an offshoot of traditional economic theory complemented by insights from psychology [17]. Briefly, behavioral economics has demonstrated how small changes in the decision environment, particularly those that align with so-called economic *rationality* by cueing individuals’ financial goals, can have powerful effects on behavioral change at both individual and societal levels [17]. In exchange for engaging in short educational quizzes on a range of public health topics, the app rewarded users with loyalty points from 4 major Canadian loyalty program providers, which can be redeemed for popular consumer products such as groceries, air travel, movies, or gas. Although monetary health incentives (eg, paying people to walk more) have shown promise with evidence of short- and long-term effects [18], only a limited amount of research has examined alternative types of financial incentives [19]. Research in consumer psychology and decision making on individuals’ responsiveness to loyalty points in particular suggests that they are overvalued by consumers in general [20] and that the way individuals behave with respect to accumulating and spending points is nonlinear [21,22]. This behavior is idiosyncratic to factors such as the effort required to earn the reward [23] and the computational ease with which individuals are able to translate points to equivalent dollar values [24]. In public health campaigns, when large financial incentives are unlikely to be suitable or sustainable [25], opportunities for more financially feasible types of incentives are worthy of further study. In addition, a robust understanding of the likely effectiveness of such programs requires a more nuanced examination of program factors such as which individuals are likely to respond to these types of interventions as well as what program design features (eg, size and timing of rewards) [26] are influential in maintaining engagement with the platform.

In the first few weeks after its launch in 2016, Carrot underwent several important program changes, resulting in a nonexperimental observational study [27] that can be examined to shed light on factors influencing engagement. Our primary objective was to examine the impact of reducing reward size on engagement to tackle competing predictions: although previous research has suggested that the size of a financial incentive is important for sustained engagement and thus behavior change [28,29], which is consistent with principles of economic rationality, other research [30] and theory in consumer psychology suggest that the magnitude of the incentive may be somewhat inelastic to size [30-32]. The secondary objectives were to examine whether reward timing, type of reward, and health intervention content influence engagement.

## Methods

### Background

Carrot Insights Inc was a private company that developed the Carrot Rewards app, in conjunction with a number of federal and provincial government partners (the federal-provincial funding arrangement is described elsewhere) [16,31]. British Columbia (BC) was the company’s founding provincial partner, and Carrot Insights Inc also partnered with 4 Canadian health charities (ie, Heart & Stroke Foundation of Canada, Diabetes Canada, YMCA Canada, BC Alliance for Healthy Living), primarily for the purpose of reviewing/approving health content delivered by the app. The marketing assets of one charity and 4 loyalty partners were also leveraged in the initial weeks of the app launching in BC with, for example, 1.64 million emails sent to the members of 3 of the 5 partners [31].

### App Registration

Carrot Rewards was made available on the Apple App Store and Google Play app stores on March 3, 2016, in both English and French (Canada’s official languages). On downloading the app, users entered their age, gender, postal code, and loyalty program card number of 1 of 4 programs of their choice (ie, movie, gas, grocery, or airline). To successfully register, users had to have entered a valid BC postal code and be aged 13 years or older (the age cutoff of the participating loyalty programs). British Columbians could download the app in 1 of 3 ways: organically (ie, finding it in the app store on their own), via an email invitation from a partner, or by using the promotional code friend referral mechanism [31].

### Intervention Overview

Once the app was downloaded and registration was completed, users were offered 1 to 2 educational health quizzes per week over the first 5 weeks after registration, each containing 5 to 7 questions related to public health priorities identified by the BC Ministry of Health—healthy eating and physical activity/sedentary behavior (3 quizzes each)—and 2 separate health risk assessments that included items from national health surveys (regarding physical activity, eating and smoking habits, alcohol consumption, mental health, and overall well-being as well as the frequency of influenza immunization). The timing, content, and order of quizzes were the same for all individuals, other than the initial quiz timing, which will be discussed in greater detail in the *Study* *Design* section. Quizzes were developed to inform and familiarize users about self-regulatory health skills [32] or stepping stone behaviors (ie, goal setting, tracking, action planning, and barrier identification), skills that have been demonstrated in the past to promote health behaviors [31]. After completing a health quiz or health risk assessment and immediately earning incentives (US $0.04 to US $1.48 depending on the length, timing, and date of completion of the quiz), users could view relevant health information on partner websites. Each health quiz or assessment was designed to take approximately 1 to 3 min to complete.

### Study Design and Participants

During the roll-out of Carrot in BC, there were 3 notable changes in the program introduced by its administrators, which provided the basis for the program variance that we explored in this study. These changes were driven largely by economic necessity rather than by theory or hypothesis testing but also presented the opportunity for a longitudinal nonexperimental observational study [27].

The first 2 changes were related to the number of points that participants could earn for completing quizzes. Specifically, during the study period following the launch of the app, there were (1) differences in the number of points offered across quizzes to compensate for differing quiz duration and timing (as demonstrated in the columns for each participant in Table 1) and (2) reductions in reward magnitude offered for the same quizzes over time (as demonstrated across in the rows for each quiz in Table 1). Owing to the unforeseen popularity of the platform and the need to manage costs within a finite budget financed by Carrot’s public sector partners, the number of reward points awarded for the completion of each quiz was reduced over time. This meant that early subscribers received more points for the initial quizzes than those who were enrolled later in the study window we examined, as demonstrated by comparing the point profiles for each sample participant in Table 1. Of note, the content of the quizzes remained invariant across time and, as mentioned previously, was informed by behavioral theories in self-regulation and habit formation [33].

The third change introduced during the evaluation period was in the number of quizzes that a participant received on the day that they registered for the app: participants who self-registered between March 3 and March 17 were awarded points for registering and were immediately offered 2 quizzes (and thus opportunities to earn reward points) on the day of registration, whereas participants who registered after March 17 were also awarded points for registering but offered only one quiz on the day of registration (as demonstrated in Table 1 by comparing the offer day for participants A and B with those noted for participants C, D, and E). This created variance in terms of both (1) the number of quizzes offered at the time of enrollment and (2) the number of opportunities to earn reward points before participants faced their first 5-day waiting period between quizzes.

Taking advantage of these program-level changes to examine their impact on app engagement and attrition, we examined quiz acceptance rates over the first 5 weeks after registration for BC residents who received the first 9 offers (initial registration plus 8 quizzes) under the launch campaign and who registered for the Carrot app between the launch (March 3, 2016) and July 21, 2016 (n=54,817), with within-subjects repeated measures for each quiz, yielding 383,719 participant-level observations.

**Table 1:** Sample profiles of points awarded for quiz completion by initial registration date (Movie Reward Program).

Quiz number and names			Participant A (joined March 9)		Participant B (joined March 16)		Participant C (joined March 23)		Participant D (joined March 30)		Participant E (joined April 6)
**1. Welcome to Carrot**											
	Day	Day 1		Day 1		Day 1		Day 1		Day 1	
	Points	100		38		25		25		17	
**2. What Does Eating a Rainbow Taste Like?**											
	Day	Day 1		Day 1		Day 1		Day 1		Day 1	
	Points	98		33		33		33		17	
**3. No Gym or Equipment Needed**											
	Day	Day 1		Day 1		Day 5		Day 5		Day 5	
	Points	165		101		58		58		33	
**4. Stand Up for Your Health**											
	Day	Day 5		Day 5		Day 10		Day 10		Day 10	
	Points	53		40		40		16		16	
**5. Carrot Health Survey 1**											
	Day	Day 10		Day 10		Day 15		Day 15		Day 15	
	Points	23		23		16		16		16	
**6. Rethink Sugary Drinks**											
	Day	Day 15		Day 15		Day 20		Day 20		Day 20	
	Points	18		18		16		16		16	
**7. The 2 Colours You Shouldn’t Eat Without**											
	Day	Day 20		Day 20		Day 25		Day 25		Day 25	
	Points	17		17		17		17		17	
**8. Is Exercise Really Like Medicine?**											
	Day	Day 25		Day 25		Day 30		Day 30		Day 30	
	Points	17		15		15		15		15	
**9. Carrot Health Survey 2**											
	Day	Day 30		Day 30		Day 35		Day 35		Day 35	
	Points	14		14		14		14		14	

### Outcome Measures

For the purpose of this research, we explored the extent to which the 2 sources of program-level variance influenced the likelihood that a participant chose to engage with a given quiz. Thus, our outcome measure was a binary measure of whether a participant chose to complete each of the 8 quizzes during the initial 5 weeks postregistration.

### Data Analyses

#### Independent Variables: Natural Experiment Factors

Although we observed the number of points earned for those who completed each quiz offer, including the change in the level of reward points offered (see Reward Points Schedule (first table) in Multimedia Appendix 1 for averages across waves), one limitation of our data is that they do not contain the number of points that were offered to participants who did not choose to complete a particular quiz offer; that is, because points awarded were based on the date of completion of a particular quiz, we only observed how many points a participant earned if they completed a particular quiz. Therefore, to explore the impact of these point changes on participants’ probability of quiz acceptance, it was necessary to impute the number of points that participants who did not complete a quiz would have been offered for the completion of a particular quiz. In this case, there were 4 important pieces of observable information that inform this data imputation: (1) the app was designed such that when it was opened, participants were shown the number of points they could earn by completing each quiz on a given date; (2) the date when a particular quiz was made available to a participant; (3) the schedule of how many points an individual *could* have earned to complete a quiz on a given date; and (4) the date of quiz completion for all participants who *completed* a particular quiz. On the basis of the assumption that participants were choosing to either complete or not complete a particular quiz and to ensure the robustness of our results, we imputed the missing observations for reward points that would have been offered to noncompleting participants in 2 ways, which are detailed in Multimedia Appendix 1. As the regression results for the 2 imputation approaches were consistent (as demonstrated in the third table in Multimedia Appendix 1–Regression Results by Imputation Methods), we reported only the results for the average days to completion imputation here.

Finally, as discussed previously, another important independent variable of interest was the combination of the differences in the timing of the quizzes and the number of reward points offered. To examine the impact of the timing structure on participants’ probability of quiz acceptance, we created a dummy variable to act as the independent variable that indicates whether or not a participant was facing their first delay between quizzes.

#### Observed Variables: Individual, Intervention, and Reward Program Factors

We also examined the influence of 4 observed variables that differed across participants or quizzes but were independent of the program-level changes described previously, which are the primary focus of this study. These were (1) self-reported demographic variables of age and gender that participants logged at the time of registration, (2) dummy variables indicating the content of each quiz (healthy eating, exercise/physical activity, and health risk assessment), and (3) a set of indicator variables for each reward program under which participants could earn points via the Carrot app. Additionally, we controlled for whether a participant completed the quiz preceding the focal quiz. This accounts for the nature of the quizzes and the likely path dependence present in intervention programs of this type (ie, the propensity to respond to a quiz is likely correlated with the decision to respond to the previous quiz).

The reward program indicator variables were potentially important observed variables for 3 reasons, and all speak to our attempts to address alternative possible explanations for observed variations in quiz participation. First, each reward program has a different level of engagement with its participants and engaged in different levels of marketing efforts promoting their partnership with Carrot. As such, the inclusion of this variable may theoretically capture the potentially different levels of promotion of the Carrot app undertaken by each reward program. Second, the enrolled participant base of each reward program has varying demographic characteristics that may not be captured in the self-reported age and gender variables described earlier. Table 2 summarizes the census-level demographics (by forward sorting area—the first 3 characters of a Canadian postal code) for each program, and although generally the bases of the programs are similar, we observed some important differences between the gas program and others in terms of socioeconomic status and lifestyle characteristics (eg, commute method, urban vs extra urban). As such, we believe that this observed variable may also capture unobserved demographic differences at the individual level. Finally, each reward program varies with respect to the earnings and redemption mechanisms of the programs. One important difference between programs is that although the actual cash value of the points awarded for each completion of each quiz remained consistent across programs, the discrete number of points varied because the dollar-to-point conversion bases of the programs were not the same. For example, for the same quiz, participants would earn the same real monetary value in points for completion, the airline and grocery programs award might be 5 points, the movie reward program might offer 10 points, and the gas program might award 100 points. As such, there was a possible numeracy effect that was captured by this observed variable. Finally, some programs offer more utilitarian rewards on redemption (eg, gas, groceries), whereas others offer more experiential redeemed rewards (eg, movies, travel), which have been demonstrated to impact individual responses to reward programs [23]. Theoretically, the inclusion of this observed variable should capture the variance associated with psychological and behavioral factors. Descriptive statistics and a correlation matrix for all measures are summarized in Table 3.

**Table 2:** Demographic characteristics of the rewards program (census-level data at the forward sorting area level).

Demographic variables	Movies	Airline	Grocery	Gas
Age (years), mean (SD)	33 (0.06)	40 (0.21)	41 (0.16)	42 (0.30)
Male, n (%)	14503 (35.40)	1797 (39.98)	1742 (21.66)	1130 (44.68)
Household income^a^ (US $), mean (SD)	52,975 (76.36)	53,600 (262.72)	52,822 (161.36)	50,616 (250.36)
Bachelor’s degree or higher, mean % (SD)	21 (0.00)	25 (0.00)	18 (0.00)	17 (0.00)
Active transport for commute, mean % (SD)	9 (0.00)	11 (0.00)	8 (0.00)	8 (0.00)
Public transit for commute, mean % (SD)	13 (0.00)	14 (0.00)	9 (0.00)	9 (0.00)
Motor vehicle for commute, mean % (SD)	77 (0.00)	73 (0.00)	82 (0.00)	82 (0.00)
Immigrant population, mean % (SD)	33 (0.00)	35 (0.00)	26 (0.00)	27 (0.00)
Aboriginal population, mean % (SD)	3 (0.00)	3 (0.00)	5 (0.00)	5 (0.00)

^a^Can $ currency converted at US $1.3.

**Table 3:** Descriptive statistics and correlation matrix.

Variables	Mean (SD)	1	2	3	4	5	6	7	8	9
1. Point change since previous quiz	0.003 (0.503)	1	N/A^a^	N/A	N/A	N/A	N/A	N/A	N/A	N/A
2. First delay in quizzes	0.143 (0.350)	0.27	1	N/A	N/A	N/A	N/A	N/A	N/A	N/A
3. Movie rewards program	0.731 (0.443)	−0.02	0.00	1	N/A	N/A	N/A	N/A	N/A	N/A
4. Airline rewards program	0.080 (0.271)	0.04	0.00	−0.49	1	N/A	N/A	N/A	N/A	N/A
5. Grocery rewards program	0.143 (0.350)	−0.01	0.00	−0.68	−0.12	1	N/A	N/A	N/A	N/A
6. Eating knowledge quiz	0.286 (0.452)	−0.23	−0.26	0.00	0.00	0.00	1	N/A	N/A	N/A
7. Exercise knowledge quiz	0.429 (0.495)	0.41	0.47	0.00	0.00	0.00	−0.55	1	N/A	N/A
8. Completed previous study	0.806 (0.395)	0.09	0.16	0.12	−0.03	−0.09	−0.04	0.11	1	N/A
9. Gender (1=male)	0.350 (0.477)	0.00	0.00	0.04	0.04	−0.11	0.00	0.00	−0.02	1
10. Age	35.116 (13.195)	0.00	0.00	−0.29	0.11	0.21	0.00	0.00	−0.05	−0.04

^a^N/A: not applicable.

### Statistical Methods

Data manipulation was conducted using SPSS version 24 (IBM Corporation), and statistical analysis was performed using the xtlogit procedure in STATA version 12.1 (Stata Corp). These random effects panel logit regression method was used to explore the impact of each of our program change variables, observed variables, and control variables on participants’ probability of quiz acceptance across the 8 quiz offers received in the 5 weeks postregistration (the outcome measure). We ran our model using the average days to quiz completion imputation procedures for the data missing from individuals who did not complete a given quiz, as described in Multimedia Appendix 1. The results of this analysis are presented in the following section.

## Results

### Engagement

**Figure 1:**
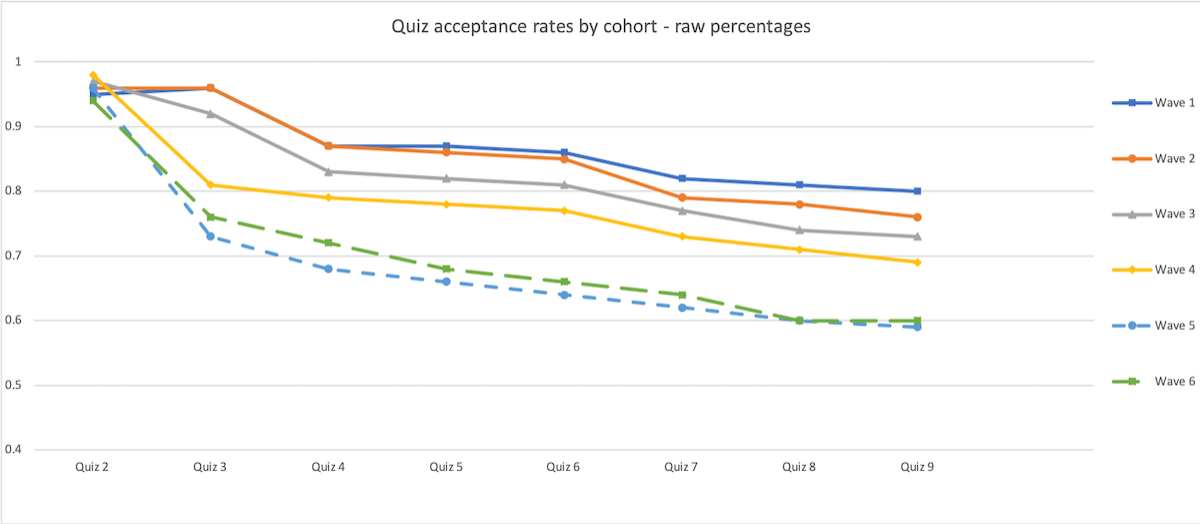
Quiz acceptance rates across quizzes by wave.

Although participants self-registered continuously throughout the study period, the date on which they registered placed them in different point schedules (see Table 1 or Reward Points Schedule (first table) in Multimedia Appendix 1 for more details). For the purpose of this study, we created analytical groups that we refer to as *waves*, which represent the point schedule in place at the time of their registration with the app. These groupings allow us to explore the effects of exogenous point changes on participant program engagement. Figure 1 demonstrates participant response rates for each of the first 8 quizzes across waves. Although not a conclusive analysis, we observed 4 key characteristics of these curves. First, the response rates to quiz 2 across the 6 waves were consistently high across all waves, ranging from 95% to 97%. This suggests that despite the significant difference in reward points offered for the completion of this quiz across waves (as demonstrated in Table 1), participants across waves are approximately equally likely to respond to this quiz. Second, response rates to the final quiz examined (quiz 9) varied, ranging from 80.5% (19,525/24,249) for wave 1 registrants to 60% (2930/4893) for wave 6 registrants, which suggests the possibility of intervening factors that predicted participant engagement between the time of initial registration and quiz 9. Third, a statistical test of the equality of the slopes from quiz 4 onward indicates that response rates across registrants in each of the waves did not differ significantly from one another after quiz 4. This suggests that the factors driving the differences in response rates appear to be independent of the underlying differences in responsiveness driven by the time of registration in the program. These results also provide support for the belief that participants were not fundamentally different from one another across the study period and indicate that it is unlikely that there were significant differences between registrants over the study period with respect to their attitudes to the underlying behavior of interest (ie, as in diffusion theories [34] where innovators, early adopters, and so on only emerge over an extended life cycle of a product). Finally, we also note that much of the decline in responsiveness across waves occurs around the time of quizzes 3 and 4, which coincides with the timing delay noted previously and which we explored in greater detail in our regression analysis.

### Regression Results

Overall, from the regression results summarized in Table 4, the regression results with respect to the features of this observational study suggest that the impact of a change in points on the likelihood of response to a quiz is positive and statistically significant (*P*<.001); however, the magnitude of the coefficient suggests that the elasticity of this response is limited. Specifically, in our data, a 10% decrease in points offered from the previous quiz resulted in a 1% decrease in the likelihood of response to a given quiz. Second, we found that the impact of the first delay that participants face was negative and statistically significant (*P*<.001). This result demonstrates that the longer delay between quizzes 2 and 3 faced by those who registered after March 17 decreased their likelihood of response by 64%.

With respect to the observed variables, we first examined the impact of demographic characteristics on the likelihood of quiz acceptance. We observed no significant effect of age (*P*=.52) on the likelihood of response; however, gender did have a significant impact (*P*<.001), whereby male participants were 15% to 16% less likely to respond to a given quiz than female participants. The next feature we explored in the model was the impact of quiz content on response rates. We observed that relative to the health risk assessments, participants were 21% more likely to respond to physical activity–related quiz offers (*P*<.001) and 10% less likely to respond to healthy eating–related quiz offers (*P*<.001). Finally, the results indicate that the reward program under which a participant registers had a significant impact on the likelihood of a quiz response. Specifically, relative to the gas rewards program, we observed that the movie rewards program participants were 355% more likely to respond to a given quiz (*P*<.001), the airline rewards program participants were 210% more likely to respond to a given quiz (*P*<.001), and grocery rewards program participants were 140% more likely to respond to a given quiz (*P*<.001).

**Table 4:** Regression results (N=383,719 observations of 54,917 individuals, with 95% CIs in parentheses).

Dependent variable=probability of quiz acceptance	Estimated coefficients (95% CI)
Point change since previous quiz	1.100 (1.065-1.137)
First delay in quizzes	0.361 (0.345-0.378)
Gender (1=male)	0.845 (0.804-0.887)
Age	0.999 (0.998-1.001)
Eating knowledge quiz^a^	0.896 (0.861-0.932)
Exercise knowledge quiz^a^	1.206 (1.156-1.257)
Movie rewards program^b^	3.552 (3.176-3.971)
Airline rewards program^b^	2.096 (1.840-2.386)
Grocery rewards program^b^	1.402 (1.247-1.575)
Completed previous study	71.891 (67.261-76.839)
Model fit—McFadden pseudo-R^2^	0.172

^a^Knowledge quiz estimates relative to health risk assessments.

^b^Rewards program estimates relative to the gasoline rewards program.

## Discussion

This study responds to a call in the literature to create a more comprehensive understanding of how contextual and program factors relate to program effectiveness [35] and, specifically, to explore how the type, amount, and timing of incentives impact program outcomes [36]. It does so by exploring how the variance induced by changes in the features of health interventions under the Carrot Rewards program (eg, incentive size, variability, quiz timing) during its BC roll-out, as well as observed differences between intervention content, reward program, and characteristics of participants, all impact the likelihood of quiz response. Thus, it contributes to the broader literature on how financial incentives [35] (particularly loyalty points) [36] and/or mHealth apps [28,37,38] can be deployed to improve uptake and engagement with health education–based interventions [37,39,40] and encourage health-related behaviors such as physical activity [18,36], management of chronic health conditions [28,41,42], smoking cessation [43,44], weight loss [45], and medication adherence and clinical treatment plans [40].

### Effectiveness

Our study demonstrates that the ongoing provision of a stream of small incentives (as low as US $0.05 per offer) can largely sustain the initial high levels of responsiveness generated by these programs. Interestingly, however, our results also demonstrate that the responsiveness to reward point decreases is relatively inelastic when compared with the other features of the interventions being offered via the Carrot app. Specifically, our results indicate that reducing the number of points associated with a particular quiz by 10% only leads to a 1% (*P*<.001) decrease in the likelihood of offer response. This relative inelasticity is consistent with the findings of Carrera et al [46], who found that US $60 for 9 gym visits in 6 weeks was no more effective than US $30 [46]. However, detailed subgroup meta-analyses by Mitchell et al [26] suggest that larger incentives can, in some cases, produce larger effect sizes in a physical activity context. As health-related interventions are often multifaceted and include incentives as just one of several components intended to produce positive health-related behavior changes, it is likely that other program features (eg, reward timing, salience of feedback, goal setting approach) can play equally important roles. Thus, when combined with previous findings that suggest that the *presence* of financial incentives matters for responsiveness [47], our results suggest that a relatively small number of points may be nearly as effective as larger numbers of points. This may be particularly true when the incentives are delivered immediately on completion of the focal behavior in question, as it leverages the formidable bias toward the present, which has been demonstrated in behavioral economics [48,49] as well as speaks to the power of technologies such as smartphones to leverage that bias for increased behavioral compliance. This nearly instantaneous connection between behavior and reward may support the potential for diminution of the size (and thus cost) of incentives necessary to produce the requisite level of behavioral change. Despite findings that suggest that there is a minimum threshold for the size of daily incentives to maintain engagement and produce the desired behavior change [25,29], recent health incentive studies appear to be using much smaller incentives in part because of the ability of mHealth apps to deliver daily rewards, consistent with behavioral theories [26].

### Contribution to Theory

In the context of the consumer psychology literature on loyalty points discussed earlier, our results support this prior work, which has demonstrated that consumers do not behave rationally (in the behavioral economics sense of the term) in their response to the quizzes and the size of the financial incentives offered. On the basis of previous research in mHealth and on the findings presented here, quiz response and thus app engagement appear to rely more on the simple presence of points (as a form of financial incentive), as opposed to the absolute quantity, as well as on the continued opportunity to earn more points without a delay. This may be for a variety of reasons. First, loyalty points already require users to accumulate over time, so there may be a built-in progress element and a recognition that the ultimate reward will be sometime in the future [22,23]. This may insulate programs such as the one discussed here from the negative effects seen in other studies where incentives get smaller over time. In addition, point programs have been hypothesized to cue goals related to gamification, where it is the acquisition of points itself that generates the affective value, rather than the economic reward per se. Our results demonstrate that the continuous, timely provision of small numbers of reward points throughout the program significantly improved retention and engagement with the Carrot app relative to previous programs that used reward points only for recruitment purposes [37] and provides support for previous findings regarding the efficiency of small, variable rewards and a decreasing schedule in maintaining engagement with health-based interventions [39,50]. Our study also provides a novel context in which to examine the behavior of individuals in response to loyalty points, but in a health context rather than a commercial context, pointing to the opportunity to use an alternative currency from which individuals already derive personal, affective value [33] as a type of financial incentive for health-related behaviors.

### Program Design Considerations

Perhaps most importantly, our results suggest that other elements of the design of these interventions need to be considered when attempting to increase response rates. Existing research has demonstrated the critical importance of including direct end user feedback in the development of such apps early in the design to ensure both short- and long-term engagement [8,40,51,52]*.* One important finding from this study is that consistent with research on habit formation [30], an increased focus is needed to retain individuals during the early engagement period. Indeed, this was the largest driver of participant attrition that we found in our study, with a differential delay between early offers being associated with a 64% decrease in response probability. This suggests that other interventions and communication initiatives should be considered during this waiting period to retain participants. The good news is that once this hurdle is surmounted (ie, once a participant re-engages after the first waiting period), retention rates between interventions are remarkably high and consistent across the registration period studied, particularly considering the small magnitude of incentives that are being offered (Figure 1).

Additional findings of our study support the importance of nonincentive elements of this program, although by and large these results are much stronger in suggesting directions for further study than they are for creating concrete recommendations for program optimization. One important factor identified in our findings with respect to participant response rates is the content of the quiz being offered. Our results suggest that when the content of the quiz is focused on healthy eating, individuals are significantly less likely to respond (10% decrease in the likelihood of acceptance relative to health risk assessments), and when the content of the quiz focused on exercise or physical activity, individuals are more likely to respond (20% increase in the likelihood of acceptance relative to health risk assessments). These results suggest that more than just the simple time cost of each intervention should be considered when designing incentive programs, perhaps incorporating individual and/or psychological factors that may influence the attractiveness of a quiz, which may increase or decrease the perceived costs of quiz acceptance and ultimately affect the propensity for uptake of an intervention.

Similarly, among the observed variables that measured age and gender, quiz content, participation in previous quiz, and reward program, we find that the strongest predictor of the likelihood of quiz acceptance among all variables studied is the point program under which a participant is earning points for completing quiz offers via Carrot. Specifically, we find that individuals who choose to earn points in the movie (355%), airline (210%), and grocery (140%) programs all have a greater likelihood of response relative to the gasoline rewards program. We previously acknowledged that the differences between these reward programs may vary along multiple dimensions, including differential promotional efforts in support of Carrot, differences in the demographic composition of the participant base for each program, differences in individual responsiveness due to a numeracy effect (ie, holding actual economic value constant, the quantity of points earned per activity varies across programs, and people respond more to higher numbers of points earned), and differences in the earning and redemption mechanisms of each point program that may each influence the responsiveness of individuals. This facet of program design requires more exploration, but our results suggest that it is yet another factor that should be considered when designing optimal incentive-based intervention programs.

### Limitations and Future Directions

One concern of the modeling approach used in this study is the possibility that the differential observed responsiveness to quizzes between what we have categorized as waves is due to underlying or inherent differences (whether demographic or psychographic) between participants who registered earlier versus later in the observed window of this study, rather than due to the previously described variations of the program. However, given (1) the very short time windows between the changes in the point scheme (as demonstrated in the first table in Multimedia Appendix 1–Reward Points Schedule), (2) the consistent slopes of the engagement curves described in the previous discussion of Figure 1, (3) the clustering approach at the participant level used in the estimation of the random effects regression model, and (4) the inclusion of a control for response to prior quizzes (which is likely to be autoregressive), we can be reasonably confident that program features drive the observed effects, rather than the unobservable characteristics of the participants. From observable data as well as theoretically, there is little to suggest that early registrants differ meaningfully from later registrants in terms of responsiveness; thus, the differences in ultimate outcomes are likely driven by features of the intervention rather than sampling considerations. Recall that participants were neither recruited nor assigned to waves; rather, those waves were our analytical construction to allow us to explore the exogenous changes to the program structure. Consequently, we would have no reason to believe that the presence of any particular individual in a given wave was anything other than random. Ideally, we would be able to more explicitly examine potential confounding factors such as individual-level differences at a psychological level (eg, promotion vs prevention orientation); regrettably, that information was not collected by the app as part of the registration process and therefore is not available.

Another limitation related to statistical analysis concerns the fact that we can only be certain of the number of points that participants saw for any given quiz for those individuals who completed the quiz in question. Although we have used conservative tests for imputation of the missing data (see Multimedia Appendix 1 for specific details), our ability to draw conclusions about individuals’ engagement is limited to those who remained engaged with the app and completed the quizzes, not about individuals who skipped or ceased participating. It is also not possible, with this study and data, to explore the extent to which individuals were aware of or sensitive to the point changes that occurred over time (eg, for participant A in Table 1, a decrease from quiz 3 of 165 points to quiz 5, where only 23 points were offered). We do find that for the individuals we were able to observe who continued to complete quizzes, even if the declining number of points was salient, it did not appear to have a large effect on engagement with the app.

We proposed several theoretical mechanisms by which different reward programs may affect engagement with the app (eg, the hedonic vs utilitarian nature of the rewards, numeracy effects), but in this study, we do not have the data to be able to empirically substantiate those hypotheses. In addition, we are unable to retroactively document the promotion or advertising of Carrot by each of the 4 participating loyalty programs or to identify underlying psychographic differences between enrollees in one program versus another. The inclusion of these factors in the model would potentially allow us to further control the reward program–specific characteristics to be able to make more informed recommendations for practitioners, program administrators, or government stakeholders in terms of the need for or effectiveness of promotion efforts to drive enrollment in and engagement with the app. In general, there is a lack of data within the literature to date to support the economic basis for the use of mHealth behavioral interventions [40,53]. Although this study does not directly tackle that issue, it does suggest an alternative dimension of cost consideration that may not be captured in existing frameworks such as the Consolidated Health Economic Evaluation Reporting Standards [53] and speaks to the need for ground effectiveness assessments in economic considerations as well as more traditional measurements such as reach. Finally, the realities of a program such as the one observed here in the variation of points offered confine us to a posthoc analysis of quizzes over a limited period. This constrains our ability to engage in repeated measures of a particular attitude or intention related to a specific context (eg, healthy eating, exercise) beyond the types of quizzes that happened to be administered within the period of observation.

Additional factors that could be integrated into the model include devising quasiexperimental studies to examine other contextual factors that may impact program engagement and effectiveness. These could include individual difference variables such as the impact of socioeconomic or health status on both app engagement and quiz effectiveness at driving attitudinal or behavioral change or program-centered factors, such as the comparative effectiveness of individual rewards versus team-based rewards, norm-based messaging, or differential offer timing. Future programming should use a modular approach such that content can be contained in a specific topic of interest to measure engagement in the quizzes in a more systematic manner and explore how the impacts of different program features may vary over time. Examining these modular programs using microrandomized controlled trial designs, similar to ongoing work by Kramer et al [54], would allow for a more targeted evaluation of how content, along with frequency, variations leads to greater and/or sustained engagement within the app. 

### Conclusions

The results of this study demonstrate that the Carrot program, built around an ongoing stream of a small number of reward points awarded instantly on completion of health interventions, was able to drive significantly higher engagement levels than demonstrated in previous literature exploring the intersection of mHealth apps and financial incentives. Furthermore, although previous studies have demonstrated that the presence of an incentive matters to user engagement, our study suggests that the number of points offered for these reward point–based health interventions is less important than other program design features such as the type of reward points being offered, the timing of intervention and reward offers, and the content of the health interventions for driving continued engagement by users.

## Appendix

Multimedia Appendix 1 Point schedule changes across study window and missing data imputation for points offered.

## Abbreviations

BC: British Columbia
mHealth: mobile health
