# Supplementary material for: Examining Responsiveness to an Incentive-Based Mobile Health App: Longitudinal Observational Study
Source: J Med Internet Res. 2020 Aug 10;22(8):e16797. doi: 10.2196/16797 (PMC7445608; doi:10.2196/16797)
Supplement: Multimedia Appendix 1 [file jmir_v22i8e16797_app1.docx]

**Multimedia Appendix 1**

**Point Schedule Changes Across Study Window**

As described in the manuscript, two program changes were related to the number of points that participants could earn for completing quizzes because of the unforeseen popularity of the platform and the need to manage costs within a finite budget financed by Carrot’s public sector partners, the number of reward points awarded for the completion of each quiz were reduced over time. Specifically, during the study period following the launch of the app there were: (1) differences in number of points offered across quizzes to compensate for differing quiz duration and timing and (2) reductions in reward magnitude offered for the same quizzes over time. Although participants self-registered continuously throughout the study period, the date on which they registered placed them in different point schedules, as demonstrated in Table 1 below.

**Table 1 –Reward Points* Schedule Offered for Quiz Completion over Time**

**(Movie Reward Program)**

| **Quiz Name and Number** | | **Schedule 1**  **(Mar 3)** | **Schedule 2**  **(Mar 14)** | **Schedule 3**  **(Mar 16)** | **Schedule 4**  **(Mar19)** | **Schedule 5**  **(Apr 5)** | **Schedule 6**  **(June 10)** |
| --- | --- | --- | --- | --- | --- | --- | --- |
| **1** | Welcome to Carrot | 100 | 74 | 38 | 25 | 17 | 8 |
| **2** | What Does Eating a Rainbow Taste Like? | 98 | 72 | 33 | 33 | 17 | 8 |
| **3** | No Gym or Equipment Needed | 165 | 131 | 101 | 58 | 33 | 17 |
| **4** | Stand Up for Your Health | 132 | 53 | 45 | 40 | 16 | 17 |
| **5** | Carrot Health Survey 1 | 130 | 37 | 37 | 23 | 16 | 8 |
| **6** | Rethink Sugary Drinks | 70 | 33 | 32 | 18 | 16 | 8 |
| **7** | The 2 Colours You Shouldn’t Eat Without | 33 | 30 | 23 | 17 | 17 | 17 |
| **8** | Is Exercise Really Like Medicine? | 36 | 30 | 17 | 17 | 15 | 8 |
| **9** | Carrot Health Survey 2 | 32 | 17 | 17 | 17 | 14 | 8 |

* Points have been indexed relative to points offered under Schedule 1, for Quiz 1 completion at the request of our partner.

**Missing Data Imputation for Points Offered**

As noted in the manuscript, although we observed the number of points earned for those who completed each quiz offer, including the change in level of reward points offered (please see Table 1 for averages across waves), one limitation of our data is that it does not contain the number of points that were offered to participants who did not choose to complete a particular quiz offer; that is, because points awarded were based on the date of completion of particular quiz, we only know how many points someone was offered if they completed a particular quiz. Therefore, in order to explore the impact of these point changes on participants’ probability of quiz acceptance, it was necessary to impute the number of points that participants who did not complete a quiz would have been offered for the completion of a particular quiz. Based on the assumption that participants were choosing to either complete or not complete a particular quiz, in order to ensure the robustness of our results, we imputed the missing observations for reward points that would have been offered to non-completing participants in the following ways:

1. The first imputation method assumed that on average, those who did not complete the survey viewed the quiz within the same time window as the average participant who did complete each quiz. Operationally, this imputation added the observed average number of days between quiz availability and quiz completion for individuals who completed each quiz (see Table 3 for summary) to the quiz offer date for those who did not complete the quiz to impute the number of points that would have been offered.
2. The second imputation was slightly more conservative than the previous method. It assumed that individuals who did not complete a quiz viewed the quiz within the same time frame that it took for the quiz to reach an 80% acceptance rate among those who completed the quiz. Operationally, this imputation created a proxy for the number of points that would have been offered to the non-completer by adding the observed number of days between quiz activation and 80% quiz completion for those who completed each quiz (see Table 3 for summary) to the date the quiz became available for those who did not complete the quiz.

**Table 2 – Number of Days Between Quiz Availability and Quiz Acceptance**

|  |  | **Wave 1** | **Wave 2** | **Wave 3** | **Wave 4** | **Wave 5** | **Wave 6** |
| --- | --- | --- | --- | --- | --- | --- | --- |
| **Quiz 3** | |  |  |  |  |  |  |
|  | Average | 1 | 1 | 1 | 2 | 3 | 3 |
|  | 80% Sat | 1 | 1 | 1 | 2 | 3 | 4 |
| **Quiz 4** | |  |  |  |  |  |  |
|  | Average | 2 | 2 | 2 | 2 | 3 | 3 |
|  | 80% Sat | 3 | 2 | 2 | 3 | 4 | 4 |
| **Quiz 5** | |  |  |  |  |  |  |
|  | Average | 2 | 2 | 2 | 3 | 3 | 3 |
|  | 80% Sat | 3 | 2 | 2 | 3 | 5 | 5 |
| **Quiz 6** | |  |  |  |  |  |  |
|  | Average | 2 | 2 | 2 | 3 | 3 | 3 |
|  | 80% Sat | 3 | 3 | 2 | 4 | 5 | 5 |
| **Quiz 7** | |  |  |  |  |  |  |
|  | Average | 3 | 3 | 3 | 3 | 4 | 3 |
|  | 80% Sat | 3 | 3 | 3 | 4 | 5 | 5 |
| **Quiz 8** | |  |  |  |  |  |  |
|  | Average | 3 | 3 | 3 | 3 | 4 | 4 |
|  | 80% Sat | 3 | 3 | 4 | 4 | 5 | 5 |
| **Quiz 9** | |  |  |  |  |  |  |
|  | Average | 3 | 4 | 3 | 4 | 4 | 4 |
|  | 80% Sat | 5 | 5 | 5 | 6 | 6 | 6 |

We also estimated the random effects panel regression model using the xtlogit procedure in STATA version 12.1 (Stata Corp) for both imputation methods. This random-effects panel logit regression method was used to explore the impact of each of our program change variables, observed variables and control variable on participants’ probability of quiz acceptance across the eight quiz offers received in the five weeks post-registration (the outcome measure), the results of which are included in Table 3 below.

**Table 3 – Regression Results for Different Imputation Methods**

| **Dependent Variable = Probability of Quiz Acceptance** | **Average Imputation** | **80% Saturation Imputation** |
| --- | --- | --- |
| **Point Change Since Previous Quiz** | 1.100 | 1.178 |
|  | (1.065, 1.137) | (1.139, 1.218) |
| **First Delay in Quizzes** | 0.361 | 0.363 |
|  | (0.345, 0.378) | (0.347, 0.380) |
| **Gender (1=male)** | 0.845 | 0.842 |
|  | (0.804, 0.887) | (0.801, 0.885) |
| **Age** | 0.999 | 0.999 |
|  | (0.998, 1.001) | (0.997, 1.001) |
| **Eating Knowledge Quiz^b^** | 0.896 | 0.894 |
|  | (0.861, 0.932) | (0.859, 0.930) |
| **Exercise Knowledge Quiz^b^** | 1.206 | 1.183 |
|  | (1.156, 1.257) | (1.134, 1.234) |
| **Movie Rewards Program^a^** | 3.552 | 3.672 |
|  | (3.176, 3.971) | (3.277, 4.115) |
| **Airline Rewards Program^a^** | 2.096 | 2.129 |
|  | (1.840, 2.386) | (1.864, 2.431) |
| **Grocery Rewards Program^a^** | 1.402 | 1.419 |
|  | (1.247, 1.575) | (1.259, 1.599) |
| **Completed Previous Study** | 71.891 | 68.473 |
|  | (67.261, 76.839) | (64.116, 73.126) |
| **Model Fit – McFadden Pseudo-R^2^** | .172 | .173 |

**N= 383,719 observations of 54,917 individuals, 95% confidence interval in parentheses.**

**^a^ rewards program estimates relative to gasoline rewards program**

**^b^ knowledge quiz estimates relative to health risk assessments**
